# Allosteric effects of chromophore interaction with dimeric near-infrared fluorescent proteins engineered from bacterial phytochromes

**DOI:** 10.1038/srep18750

**Published:** 2016-01-04

**Authors:** Olesya V. Stepanenko, Mikhail Baloban, Grigory S. Bublikov, Daria M. Shcherbakova, Olga V. Stepanenko, Konstantin K. Turoverov, Irina M. Kuznetsova, Vladislav V. Verkhusha

**Affiliations:** 1 Laboratory of Structural Dynamics, Stability and Folding of Proteins, Institute of Cytology, Russian Academy of Sciences, St. Petersburg 194064, Russian Federation; 2 Department of Anatomy and Structural Biology, Albert Einstein College of Medicine, Bronx, New York 10461, USA; 3 Department of Biophysics, Peter the Great St. Petersburg Polytechnic University, St. Petersburg 194064, Russian Federation; 4 Department of Biochemistry and Developmental Biology, Faculty of Medicine, University of Helsinki, Helsinki 00290, Finland

## Abstract

Fluorescent proteins (FPs) engineered from bacterial phytochromes attract attention as probes for *in vivo* imaging due to their near-infrared (NIR) spectra and use of available in mammalian cells biliverdin (BV) as chromophore. We studied spectral properties of the iRFP670, iRFP682 and iRFP713 proteins and their mutants having Cys residues able to bind BV either in both PAS (Cys15) and GAF (Cys256) domains, in one of these domains, or without these Cys residues. We show that the absorption and fluorescence spectra and the chromophore binding depend on the location of the Cys residues. Compared with NIR FPs in which BV covalently binds to Cys15 or those that incorporate BV noncovalently, the proteins with BV covalently bound to Cys256 have blue-shifted spectra and higher quantum yield. In dimeric NIR FPs without Cys15, the covalent binding of BV to Сys256 in one monomer allosterically inhibits the covalent binding of BV to the other monomer, whereas the presence of Cys15 allosterically promotes BV binding to Cys256 in both monomers. The NIR FPs with both Cys residues have the narrowest blue-shifted spectra and the highest quantum yield. Our analysis resulted in the iRFP713/Val256Cys protein with the highest brightness in mammalian cells among available NIR FPs.

The development of near-infrared (NIR) fluorescent proteins (FPs) from bacterial phytochromes (BphPs) has substantially progressed recently because of the great need for genetically encoded NIR probes to noninvasively study metabolic processes deep within the tissue of mammals^1^,^2^. Compared with visible light, NIR light has the advantages of more deeply penetrating mammalian tissues and resulting in less light scattering. The probes that have been recently developed from BphPs include permanently fluorescent NIR FPs^3^,^4^,^5^,^6^,^7^, photoactivatable NIR FPs^8^ and NIR reporters of protein-protein interactions^9^,^10^.

BphPs consist of a photosensory core module and an output effector module, which is typically a histidine kinase^11^,^12^. The photosensory module is formed by PAS (Per-ARNT-Sim repeats), GAF (cGMP phosphodiesterase/adenylate cyclase/FhlA transcriptional activator), and PHY (phytochrome-specific) domains. For light absorption BphPs covalently incorporate a heme-derived linear tetrapyrrole, biliverdin IXα (BV), as a chromophore^13^. The chromophore binding occurs via a thioether bond formed between pyrrole ring A of BV and a conserved Cys residue located in the N-terminal extension of the PAS domain^14^,^15^. In contrast to BphPs, plant and cyanobacterial phytochromes covalently bind other bilin chromophores called phytochromobilin (PΦB) and phycocyanobilin (PCB), respectively; these chromophores have less extended conjugated electron systems^16^,^17^. The plant and cyanobacterial phytochrome PΦB and PCB both covalently bind to a conserved Cys residue located in their GAF domain^18^,^19^.

BphPs exist in two stable states that absorb at 680–710 nm (the Pr state or red-absorbing state) and 740–760 nm (the Pfr state or far red-absorbing state). The ground Pr state (or Pfr state) may be converted into the Pfr state (or Pr state) upon illumination with red (or far-red) light, respectively. Engineering of BphPs into permanently fluorescent NIR FPs requires stabilization of the Pr state of the BV chromophore, destabilization of its Pfr state, and disruption of the hydrogen bond network between BV and its microenvironment^1^,^2^,^20^. These conditions are achieved by the deletion of the PHY and effector domains and by the introduction of amino acid substitutions into the immediate environment of the chromophore. This strategy was employed to develop several permanently fluorescent NIR FPs from the PAS-GAF domains of *Deinococcus radiodurans Dr*BphP^4^,^5^,^6^ and five multicolor iRFP proteins from *Rhodopseudomonas palustris Rp*BphP2 and *Rp*BphP6^3^,^7^. Unlike *Dr*BphP-based NIR FPs, the series of iRFP proteins efficiently incorporated the endogenous BV chromophore, which is abundant in mammalian tissues.

More interestingly, iRFP670, which is engineered from *Rp*BphP6, and iRFP682, which is engineered from *Rp*BphP2, exhibited ~30-35 nm blue-shifted absorbance and fluorescence, which is unusual for other BphP-derived NIR FPs. An analysis of the amino acid sequence of iRFP670 and iRFP682 showed an additional Cys residue in the GAF domain. This Cys is not present in other NIR FPs proteins or in any BphPs, all of which only contain the common BV-binding Cys in the PAS domain. Our analysis of the *Rp*BphP2 crystal structure^21^ showed that this Cys is localized in the BV-binding pocket of the GAF domain in the conserved SP*X*H amino acid motif of BphPs, in which the *X* residue was replaced with Cys256 (here and below the amino acid numbering is in accordance with the alignment in Supplementary Fig. 1). This residue is located at a position analogous to that of the conserved Cys residue in the GAF domain of plant and cyanobacterial phytochromes^12^,^22^. We speculated that this Cys residue in iRFP670 and iRFP682 might be involved in BV binding and might result in the unusual spectral blue shift of these proteins.

In this paper, to characterize iRFP670 and iRFP682 in more detail, we generated a range of mutants with Cys residues in either the PAS or GAF domains and mutants lacking the Cys residues. We generated similar mutants of iRFP713, which were engineered in a manner similar to that for iRFP682 from *Rp*BphP2. In addition, for iRFP713, we substituted the amino acid at the 204 position in the conserved PXS*D*IP region. As known, the substitution of the conserved Asp204 of wild-type BphPs disrupts the Pr to Pfr photoconversion; moreover, this substitution is necessary to stabilize the Pr state in NIR FPs^23^. The spectral characteristics of the iRFP670, iRFP682 and iRFP713 mutants were evaluated in buffer and with denaturing reagent. Analysis of the data revealed an allosteric effect in the dimeric structure of iRFP variants on the binding of BV to Cys residues in the second monomer. We also showed that the iRFP713 mutant with Cys residues in both the PAS and GAF domains exhibits the highest molecular brightness and quantum yield among all BphP-derived FPs. Moreover, the effective brightness of this mutant in mammalian cells was 50% higher than that of iRFP682 and iRFP713 and 30% higher than the second brightest BphP-derived FP, iRFP670. Due to the blue spectral shift, this new bright NIR FP was used for two-color imaging in live mammalian cells.

## Results and Discussion

### BV binding to iRFP713, iRFP682, iRFP670 and their mutants

The dimeric NIR FPs iRFP682 and iRFP670 exhibit blue-shifted (~30–45 nm) absorbance and fluorescence spectra compared with those of the dimeric NIR FP iRFP713 (Table 1). The distinctive feature of iRFP682 and iRFP670 is the presence of Cys256 in the GAF domain in addition to the canonical for BphPs Cys15 in the PAS domain of each protein monomer (Supplementary Fig. 1). To identify the origin of the substantial difference in the spectral characteristics of these NIR FPs, we made the following mutants of iRFP713, iRFP682 and iRFP670: with Cys15 residue only in the PAS domain (iRFP670/C256S, iRFP682/C256S and iRFP713), with Cys256 residue only in the GAF domain (iRFP670/C15S, iRFP682/C15S and iRFP713/C15S/V256C), with Cys residues in both the PAS and GAF domains (iRFP670, iRFP682 and iRFP713/V256C), and without the Cys residues (iRFP670/C15S/C256S, iRFP682/C15S/C256S and iRFP713/C15S). After expression in *E.coli*, all these NIR FP variants were colored, suggesting that all of them efficiently incorporated the BV chromophore. We then applied a zinc staining assay to study the nature of the BV incorporation.

As expected, the NIR FP variants with the BphP-conserved Cys15 residue in the PAS domain were able to covalently bind BV as detected with zinc staining (Supplementary Fig. 2). Moreover, the zinc staining indicated that BV was also covalently bound to NIR FP variants with Cys256 residue in the GAF domain. This result was not surprising because although the Cys15 and Cys256 residues are located far from each other in the polypeptide, these two residues are spatially located near the reactive C3^2^ atom of the A ring of BV in the pocket of the GAF domain. Furthermore, Cys256 is known to be the conserved chromophore binding residue in cyanobacteriochromes^12^,^22^. The NIR FP variants containing both Cys15 and Cys256 were detected by zinc staining, whereas the NIR FP variants without these Cys residues were not. Interestingly, while both the coomassie blue and the zinc staining show single bands for the NIR FP variants containing Cys residues either in the PAS or in the GAF domain, the variants containing both Cys15 and Cys256 (iRFP670, iRFP682 and iRFP713/V256C) exhibit two close bands (Supplementary Fig. 2). The reason for this band splitting is not clear. The gel filtration of the NIR FP variants with two Cys residues showed the single elution peaks (Supplementary Fig. 3) that confirmed the absence of impurities in these protein samples.

### Characteristics of NIR FPs with different Cys contents and location

**Figure 1:**
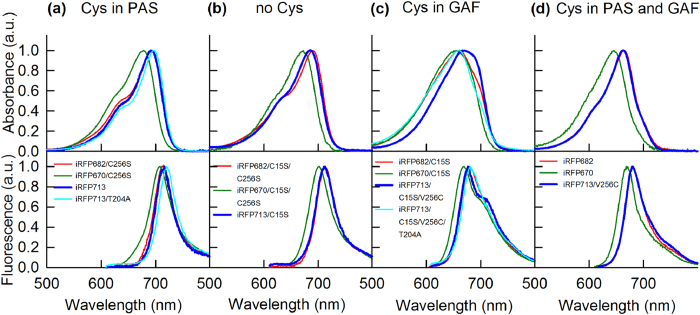
Spectral properties of iRFP713, iRFP682 and iRFP670 variants. Absorbance (top row) and fluorescence emission (bottom row) spectra of the iRFP713, iRFP682 and iRFP670 variants with the Cys residue either (**a**) in the PAS domain or (**c**) in the GAF domain, (**b**) without Cys15 and Cys256, or (**d**) with the Cys residues in both the PAS and GAF domains. Fluorescence emission was excited at 600 nm.

**Figure 2:**
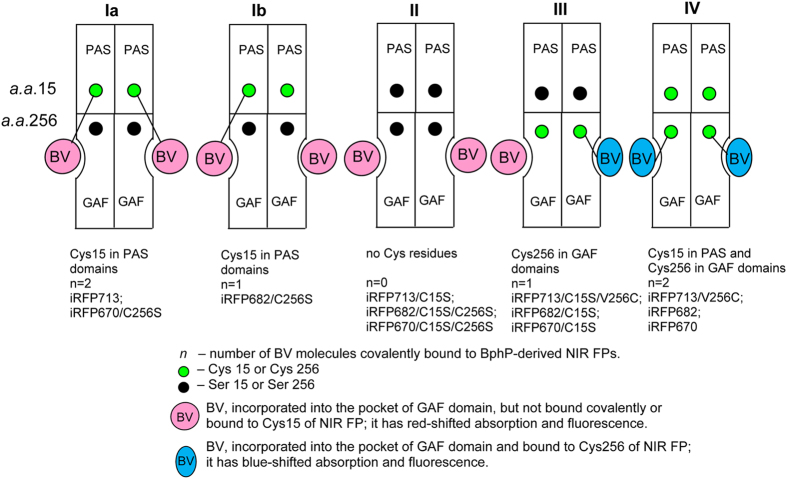
Proposed allosteric model for BV interaction with monomers in dimeric NIR FPs. In NIR FPs with Cys residue only in the PAS domain, either binding of BV to Сys15 occurs in both monomers of the dimeric protein (as in iRFP713 and iRFP670/C256S) (**Ia**) or BV binding in one monomer of the dimeric protein prevents the covalent binding of BV to Сys15 in the other monomer (as in iRFP682/C256S) (**Ib**). (**II**) No covalent BV binding occurs in NIR FPs without Cys residues in the PAS and GAF domains. (**III**) In NIR FPs with Cys residue only in the GAF domain, binding of BV to Сys256 in one monomer of the dimeric protein prevents the covalent binding of BV to Сys256 in the other monomer. (**IV**) In NIR FPs with Cys residues in both the PAS and GAF domains, Cys15 promotes the covalent binding of BV to Cys256 in both monomers.

We next examined the absorption and emission spectra of the iRFP713, iRFP682 and iRFP670 variants with different locations and numbers of Cys residues (Supplementary Fig. 4). The spectral properties were not specific to NIR FPs but depended on the presence and location of the Cys residues in their PAS and/or GAF domains. We divided the NIR FPs into four groups based on their spectral properties (Table 1, Figs 1 and 2): NIR FP variants having Cys residue only in PAS domain (Cys15) (group I), variants without Cys residues suitable for the covalent binding of BV (group II), variants with Cys residue only in the GAF domain (Cys256) (group III), and variants containing both Cys15 and Cys256 in the PAS and GAF domains (group IV).

The mutant variants of the studied proteins bearing Cys residue only in the PAS domain (iRFP713, iRFP713/T204A, iRFP682/С256S and iRFP670/С256S) had the most red-shifted absorption and fluorescence (Fig. 1(a), Table 1(group I)). The absorption and fluorescence spectra of variants containing no Cys residues capable of BV binding (iRFP713/C15S, iRFP682/C15S/С256S and iRFP670/C15S/С256S) were similar to the spectra of the proteins with the Cys residue only in the PAS domain. iRFP713/C15S and iRFP670/C15S/С256S had the slightly blue-shifted absorption and fluorescence spectra with respect to their counterparts from group I (Fig. 1(b), Table 1(group II)), whereas iRFP682/C15S/С256S had the slightly blue-shifted absorption spectrum and the fluorescence spectrum that is almost identical to that of iRFP682/C256S (Fig. 1(b), Table 1(group II)). These data suggest that both the covalently bound and the noncovalently incorporated BV molecules contribute to the spectral characteristics of NIR FPs (Fig. 1(a,b)). The π-electron system of a BV molecule incorporated into the protein pocket might be highly similar to that of BV covalently linked to Cys15, although comparison of the fluorescence quantum yields of these proteins indicated a difference in the mobility of the covalently and the noncovalently bound BVs. BV incorporated in the GAF pocket can likely adopt two conformations (see below), and the covalent binding likely immobilizes one of these isoforms.

Within the NIR FP groups I and II (Table 1), the lowest fluorescence quantum yield is exhibited by the iRFP682 mutants (iRFP682/C256S and iRFP682/C15S/C256). A previous study showed that BV was incorporated into iRFP713 at a molar ratio of 1:1^24^, suggesting that the BV binding to one iRFP713 monomer did not interfere with the BV binding to the other monomer. Therefore, the observed iRFP682/C256S and iRFP682/C15S/C256 characteristics may indicate that the covalent binding of BV to the first monomer allosterically affects the ability of the second monomer to bind BV. This assumption was confirmed in the GdnHCl-induced protein denaturation experiments below.

All NIR FP variants containing Cys256, regardless of whether they do (iRFP713/V256C, iRFP682 and iRFP670) or do not (iRFP713/C15S/V256C, iRFP713/C15S/V256C/T204A, iRFP682/С15S and iRFP670/С15S) have Cys15 in the PAS domain, showed substantially blue-shifted absorption and fluorescence (Fig. 1(c,d), Table 1(groups III, IV)). Thus, the spectra of these variants are mainly determined by BV covalently bound to Cys256 in the GAF domain. The blue-shift of the chromophore covalently bound to Cys256 may be caused by a change in the degree of the π-electron conjugation relative to that for the BV covalently linked to canonical Cys15.

It would be natural to assume that NIR FPs carrying only Cys256 in the GAF domain would have narrower absorption and fluorescence spectra compared to those of the proteins having both the Cys15 and Cys256 residues. In the latter case, one could expect the formation of both chromophore adducts: one characterized by blue-shifted and another by red-shifted spectra, because two putative BV binding sites are present. This scenario eventually could lead to a broadening of the spectra of NIR FP variants with both Cys256 and Cys15. However, the experimental data show the opposite effect.

The NIR FP variants with Cys256 only in the GAF domain exhibit the obvious red-shifted shoulders in their absorption and fluorescence spectra (Fig. 1(c,d), Supplementary Fig. 4). As a result, the NIR FP variants with Cys256 only (Table 1(groups III)) have the broadened absorption and fluorescence spectra with a large full width at half maximum (FWHM). In contrast, the NIR FP variants with the Cys residues in both domains have the narrow blue-shifted absorption and fluorescence spectra (Table 1(group IV)). These findings indicate that the NIR FP variants with the Cys residue in the GAF domain contain another form of the chromophore, in addition to the blue-shifted chromophore covalently bound to Cys256. The other chromophore has the red-shifted absorption and fluorescence. The NIR FP variants with the Cys residues in the PAS and GAF domains contain only the chromophore form covalently bound to Cys256. Thus, even when both Cys15 and Cys256 are available, BV preferentially binds to Cys256. Because covalent binding of BV to a Cys residue is an irreversible process, the preferred binding of BV to Cys256 in the NIR FP variants with the Cys residues in both PAS and GAF domains is likely determined by binding kinetics: the rate of the BV binding to Cys256 significantly exceeds the rate of the BV binding to Cys15.

The spectral data of studied NIR FPs can be explained as follows: the covalent binding of BV to Сys256 in one monomer of dimeric NIR FPs bearing only the Cys256 residue allosterically inhibits the BV binding to Сys256 of the other monomer. As a result, the second monomer of these proteins holds a BV molecule that is chemically unbound to any Cys residue; this BV exhibits red-shifted absorption and fluorescence (Fig. 1(b)). This proposed model of the allosteric BV interaction with dimeric NIR FPs is presented in Fig. 2.

### Protein stability of NIR FPs and their Cys mutants

The allosteric model of the BV interaction with dimeric NIR FPs (Fig. 2) was further confirmed by measuring the spectral characteristics of iRFP713, iRFP682 and iRFP670 variants in the presence of a denaturing agent, GdnHCl. The spectral characteristics of the variants were compared to the values of the F_N_ parameter at different GdnHCl concentrations (see Methods).

**Figure 3:**
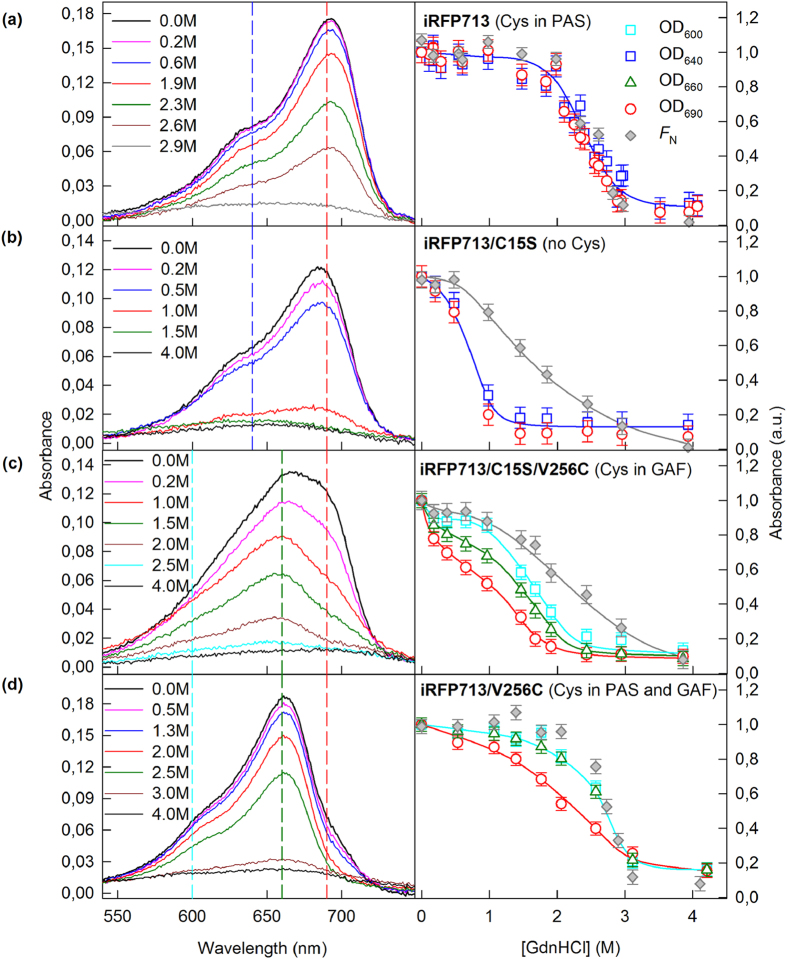
Change in the visible absorption of iRFP713 variants in the presence of GdnHCl. The absorption spectra of mutant proteins at different denaturant concentrations are presented in the left panels. The numbers on the curves indicate the denaturant concentration of the protein samples. Colored vertical dashed lines show the wavelengths selected for further analysis. In the right panels, the dependences of the optical densities at 640 and 690 nm, or those at 600, 660 and 690 nm, on the GdnHCl concentration are shown. These data were normalized to the absorption at the corresponding wavelength of the iRFP713 variant in buffered solution (n = 3; error bars are s.e.m.). The stability of the protein structure against GdnHCl-induced unfolding is shown as the dependences of the fraction of native molecules *F*_N_ on the GdnHCl concentration (gray symbols). *F*_N_ was calculated on the basis of the ellipticity at 222 nm.

**Figure 4:**
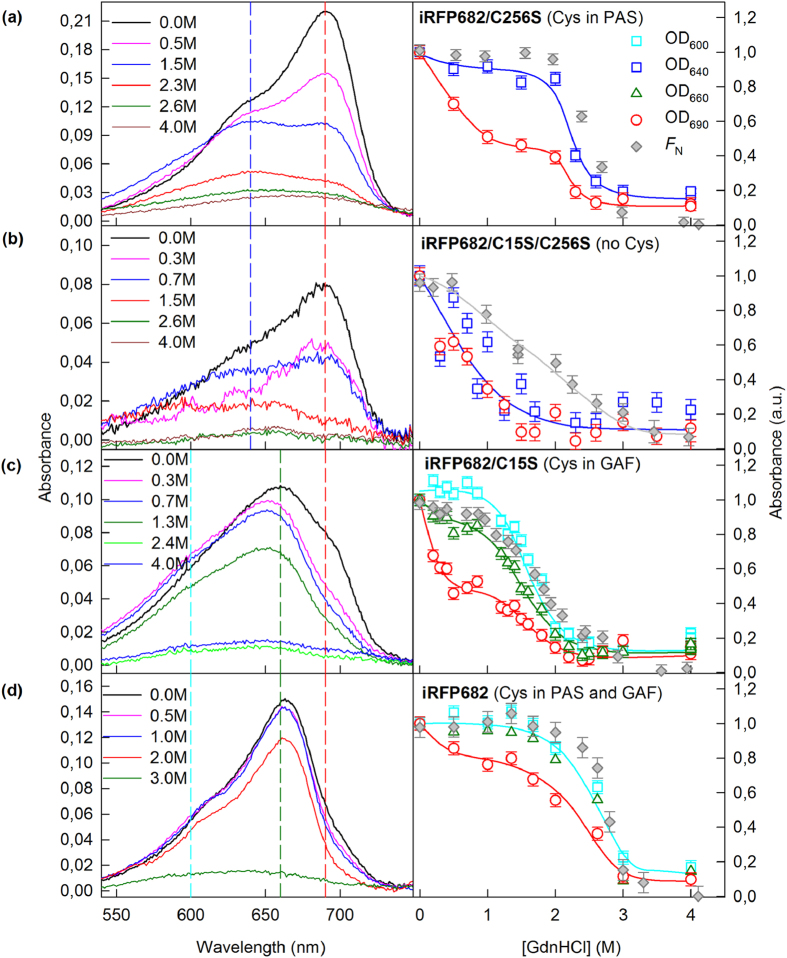
Change in the visible absorption of iRFP682 variants in the presence of GdnHCl. The absorption spectra of mutant proteins at different denaturant concentrations are presented in the left panels. The numbers on the curves indicate the denaturant concentration of the protein samples. Colored vertical dashed lines show the wavelengths selected for further analysis. In the right panels, the dependences of the optical densities at 640 and 690 nm, or those at 600, 660 and 690 nm, on the GdnHCl concentration are shown. These data were normalized to the absorption at the corresponding wavelength of the iRFP682 variant in buffered solution (n = 3; error bars are s.e.m.). The stability of the protein structure against GdnHCl-induced unfolding is shown as the dependences of the fraction of native molecules *F*_N_ on GdnHCl concentration (gray symbols). *F*_N_ was calculated on the basis of ellipticity at 222 nm.

**Figure 5:**
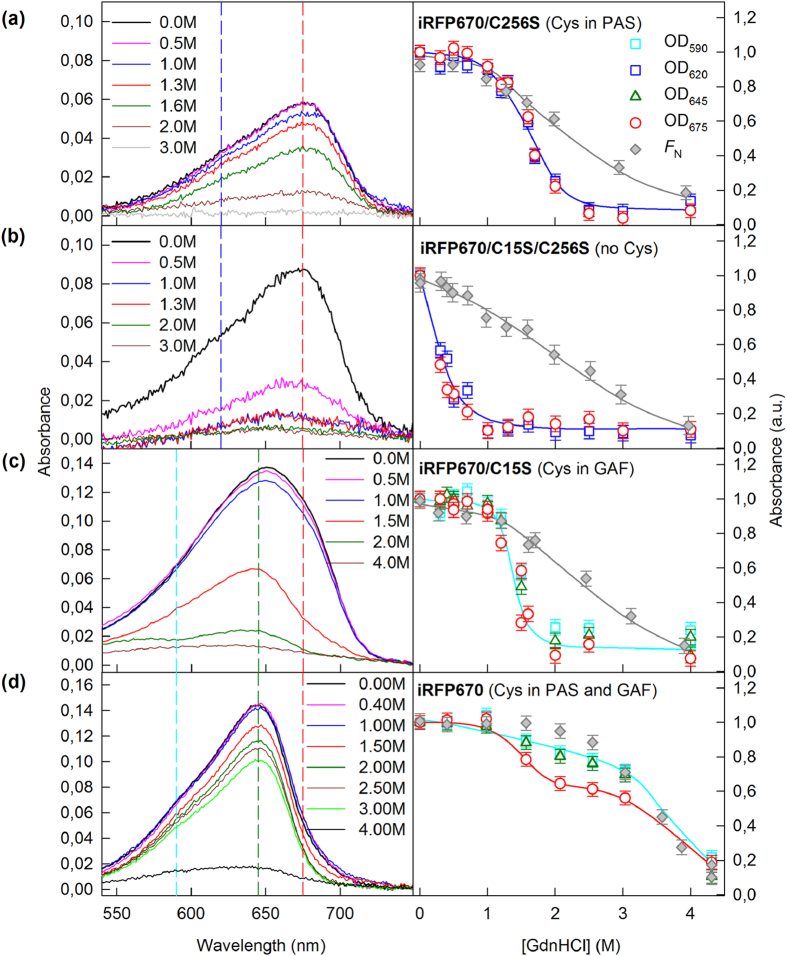
Change in the visible absorption of iRFP670 variants in the presence of GdnHCl. The absorption spectra of mutant proteins at different denaturant concentrations are presented in the left panels. The numbers on the curves indicate the denaturant concentration of the protein samples. Colored vertical dashed lines show the wavelengths selected for further analysis. In the right panels, the dependences of optical densities at 620 and 675 nm, or those at 590, 645 and 675 nm, on the GdnHCl concentration are shown. The data were normalized to the absorption at the corresponding wavelength of the iRFP670 variant in buffered solution (n = 3; error bars are s.e.m.). The stability of the protein structure against GdnHCl-induced unfolding is shown as the dependences of the fraction of native molecules *F*_N_ on GdnHCl concentration (gray symbols). *F*_N_ was calculated on the basis of ellipticity at 222 nm.

BV was the most weakly associated with NIR FP variants having no Cys residues capable of covalent BV binding (iRFP713/C15S, iRFP682/C15S/C256S and iRFP670/C15S/C256S) (Figs 3(b), 4(b), 5(b) and 2(panel II)). In these variants, the chromophore dissociated even at minimal GdnHCl concentrations where the native protein structure was still preserved (Figs 3(b), 4(b) and  5(b)). These variants had the lowest quantum yields and the highest p*K*a values among all studied NIR FPs (Table 1). Remarkably, the stability of the structure of these proteins was comparable to that of the apoforms (Supplementary Fig. 7, Supplementary Table 1). In other words, the noncovalent incorporation of BV into the pocket of the GAF domain did not stabilize the protein structure.

In variants of iRFP713 that contain the Cys15 residue only in the PAS domain (iRFP713, iRFP713/T204A), both monomers presumably attach to the chromophore covalently (Fig. 2(panel Ia)); these variants possessed a much more stable structure: the chromophore release occurred concomitantly with the protein structure disruption (Fig. 3(a), Supplementary Fig. 6(a), Supplementary Table 1). The covalent binding of the chromophore in the iRFP670 variant containing the Cys15 residue only in the PAS domain (iRFP670/C256S) also contributed to the strength of the protein-BV interaction, but BV molecules left the chromophore pocket of the GAF domain at lower GdnHCl concentrations than the concentrations for protein denaturation (Fig. 5(a)). At the same time, iRFP670/C15S and iRFP670/C256S had similar stability of the protein structure, which was comparable to that of the iRFP670 apoform (Fig. 5(a), Supplementary Fig. 7, Supplementary Table 1). This finding meant that a covalent bond between any Cys residue and BV did not stabilize the protein and could indicate a weaker BV-protein interaction in the iRFP670 variants than in the iRFP713 and iRFP682 variants.

A specific behavior of iRFP682/C256S, which only has Cys residue in the PAS domain similarly to iRFP713 and iRFP670/C256S, is notable. The dependences of absorbance, measured at the maximum and at the long-wavelength edge of the spectrum during GdnHCl-induced unfolding of iRFP682/C256S, differed substantially, suggesting that this variant contained two chromophore species. The first chromophore species was readily dissociated from the GAF pocket of iRFP682/C256S at GdnHCl concentrations less than 1 M, similar to the findings for NIR FP variants that had no Cys residues capable of BV binding. The second chromophore species was released from the GAF pocket of iRFP682/C256S only during disruption of the protein structure (Fig. 4(a)). From these observations, we assumed that the first chromophore species corresponds to the noncovalently incorporated BV (Fig. 2(panel Ib)). The presence of a noncovalently incorporated chromophore in iRFP682/C256S also explains the lower quantum yield of this protein compared to that of the iRFP670 and iRFP713 variants with Cys residue in the PAS domain (Table 1).

The iRFP713 and iRFP682 variants that contain Cys residue in the GAF domain covalently bind to a single BV molecule through Cys256 in one monomer, and the second BV is noncovalently incorporated in a pocket of the other monomer (Fig. 2(panel III)). The absorbance at the long-wavelength edge of the spectrum decreased at as low GdnHCl concentration (<1 M), as in the case of NIR FP variants containing no Cys residues capable of BV binding (Figs 3(c) and  4(c), Supplementary Fig. 6(b)). The dependences of the absorbance measured at the maximum and at the long-wavelength edge of the spectrum during GdnHCl-induced unfolding of iRFP670/C15S were effectively the same, likely because of the diverse BV-protein interactions in the iRFP670 variants compared to the iRFP713 and iRFP682 variants.

Dimeric NIR FPs bearing Cys15 and Cys256 in the PAS and GAF domains, which covalently bind BV molecules via Cys256 in both monomers (Fig. 2(panel IV)), had increased protein stability compared to protein variants with Cys15 in the PAS domain (Figs 3(d), 4(d) and  5(d), Supplementary Fig. 7, Supplementary Table 1). This effect was more pronounced for iRFP670. For iRFP713/V256C and iRFP682, the introduction of the Cys256 residue in the GAF domain in addition to the Cys15 residue in the PAS domain slightly stabilized the proteins. Possibly, the NIR FPs bearing Cys15 and Cys256 in the PAS and GAF domains bind BV more strongly, resulting in a higher fluorescence quantum yield and molecular brightness (Table 1). Importantly, the highest quantum yield of 14.5% and the highest molecular brightness among the currently available BphP-derived NIR FPs^2^ was observed for the iRFP713/V256C variant (Table 1).

We next analyzed the effect of substitutions at position 204 on the spectral characteristics of iRFP713 variants having Cys residues only in either the PAS or GAF domains. The Ala substitution of Thr204 in iRFP713, which is near the chromophore, only caused a slight red-shift in the absorption and fluorescence (Fig. 1(a), Table 1). This result was in contrast to the introduction of the Cys residue at position 256, which is farther from the chromophore, resulting in a spectral shift of ~30 nm. In the iRFP713 variants that only have the Cys256 residue in the GAF domain, the residue at position 204 affected the ratio of the blue-shifted species that is covalently bound to Cys256 relative to the red-shifted noncovalently incorporated chromophore species (Fig. 1(c)). NIR FPs containing Thr204 had more pronounced absorption and fluorescence of the red-shifted noncovalently incorporated chromophore compared to the proteins containing Ala204.

### Structure of chromophore covalently bound to Cys256

The spectral data and the zinc staining indicated that BphP-derived NIR FPs are capable of covalent binding of BV chromophore via both Cys15 in the PAS domain and Cys256 in the GAF domain. According to X-ray data of the PAS-GAF domains of *R*pBphP2 bacterial phytochrome^21^, which was used as a template for engineering iRFP682 and iRFP713, the C3 atom of ring A of BV and the C3^1^ and C3^2^ atoms of the side chain of ring A are located at distances of 3.9, 3.0 and 1.6 Å, respectively, from the thioether bond-forming sulfur atom SG of Cys15^25^. The crystal structure indicates that BV is associated with Cys15 through a covalent bond between the SG atom of the Cys residue and C3^2^, which is the closest BV atom to SG. We also determined the BV-Cys256 distance, assuming that the SG atom of Cys256 will have the same localization as the CG1 (the most proximal to BV) atom of Val256 in *R*pBphP2 (Supplementary Fig. 5(a)). This analysis showed that ring A of BV is the closest to the residue at position 256. The distances between the SG atom of Cys256 and the С3, C3^1^ and C3^2^ atoms of the chromophore are 3.6, 3.4 and 4.6 Å, respectively. Thus, the SG atom of Cys256 might covalently bind to one of the most proximal to SG atoms of BV, C3^2^ or C3^1^.

A previous study showed that in the cyanobacterial phytochrome Cph1, the formation of the covalent bond between its chromophore and Cys259 is preceded by the chromophore entering the GAF domain pocket^26^. We propose that the same mechanism is valid for BphPs. Our experimental data showing that NIR FP variants without Cys15 and Cys256 residues are able to form fluorescent complexes with BV confirm this scheme of BV association. The π-electron system of free BV (Supplementary Fig. 5(b)) is not identical to the system of BV covalently bound via Cys15 (Supplementary Fig. 5(с)), but the degrees of π-electron conjugation in both cases are almost the same. Thus, the variants containing no Cys residues capable of BV binding and the proteins that only have Cys residue in the PAS domain have similar spectral characteristics and are spectrally shifted by no more than 4–7 nm.

We suppose that the BV that has entered the GAF domain pocket has some conformational flexibility. Fluctuations of the side chain of BV ring A may bring it near the side chains of residues at positions 15 and 256. In the NIR FP variants containing both Cys15 and Cys256 residues, a covalent bond between Cys256 and BV is preferentially formed, possibly because the product of this reaction is kinetically favored compared to the BV adduct bound to SG of Cys15. The substantial blue shift of the absorption and fluorescence of BV that is covalently bound to Cys256 confirms that the chemical structure of this BV adduct is different from that of BV covalently bound to Cys15. For the former, the degree of the π-electron conjugation in the chromophore system is decreased. We suppose that this decrease could be achieved by moving the position of the double bond from ring A out of the π-conjugated system of the BV chromophore (Supplementary Fig. 5(d, e)) while maintain the total number of double bonds in the BV adduct. A similar mechanism of tetrapyrrole chromophores isomerization has been proposed for several cyanobacteriochromes^27^,^28^. The proposed structures of the BV adducts covalently bound to Cys256 was recently confirmed by the X-ray analysis of a monomeric BphP1-FP/C20S NIR FP engineered from *Rp*BphP1 of *Rhodopseudomonas palustris*, which has the Cys residue in the GAF domain^29^. It was shown that in the BphP1-FP/C20S the BV binding to Cys256 can occur either via its C3^2^ atom or via its C3^1^ atom, thus, resulting in two BV adduct chromophores.

### NIR FP brightness and two-color imaging in mammalian cells

The biophysical parameters of iRFP670, iRFP682 and iRFP713 variants differed; thus, we were curious about their properties in mammalian cells. We cloned all iRFP variants into a mammalian expression vector and transiently transfected HeLa cells. Flow cytometry analysis showed that the iRFP670/C256S- and iRFP682/C256S-expressing cells had the highest effective (i.e., cellular) brightness among the mutants; these values were respectively 86% and 84% compared to the parental iRFP670 and iRFP682 and 103% and 88% compared to the control iRFP713 (Table 1, Supplementary Fig. 8). Despite having higher quantum yields than iRFP670/C256S and iRFP682/C256S, the iRFP670/C15S- and iRFP682/C15S-expressing cells were 3-fold dimmer. Almost no NIR fluorescence was detected for cells expressing iRFP670/C15S/C256S and iRFP682/C15S/C256S (Table 1, Supplementary Fig. 8).

**Figure 6:**
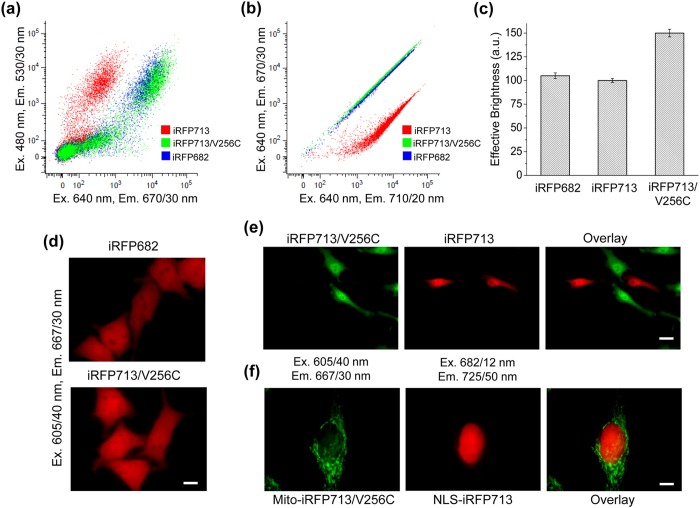
Brightness and two-color detection of mammalian cells expressing iRFP682, iRFP713 and iRFP713/V256C variants. (**a**) HeLa cells were co-transfected with one of the iRFP variants and EGFP as the transfection efficiency control and were analyzed in a NIR channel (x axis: excitation at 640 nm and emission at 670/30 nm) and green channel (y axis: excitation at 488 nm and emission at 530/30 nm) using flow cytometry. (**b**) HeLa cells transiently expressing one of the iRFP variants were analyzed using a flow cytometer with a 640 nm laser and the combination of 670/30 nm (y axis) and 710/20 nm (x axis) emission filters. For each cell type, 50,000 events were analyzed. (**c**) The NIR fluorescence of HeLa cells was normalized to the EGFP intensity and to the excitation and emission spectra of each iRFP variant. The normalized fluorescence intensity of the cells expressing iRFP713 was assumed to be 100%. (**d**) Representative images of HeLa cells transfected with iRFP682 (top) and iRFP713/V256C (bottom) are shown. The 605/40 nm excitation and 667/30 nm emission filters were used. Equal exposure times were used for all cells. Scale bar, 10 μm. **(e)** Two-color imaging of co-cultured live HeLa cells cytoplasmically expressing either iRFP713/V256C (green pseudocolor) or iRFP713 (red pseudocolor). Scale bar, 25 μm. **(f)** Two-color imaging of live HeLa cells co-transfected with iRFP713/V256C (green psedocolor) fused to a mitochondrial targeting signal (Mito) and iRFP713 (red pseudocolor) fused to a nuclear localization signal (NLS). Scale bar, 10 μm. In **(e, f)** each cell was imaged in the 605/40 nm excitation and 667/30 nm emission channel (presented in green pseudocolor), and in the 682/12 nm excitation and 725/50 nm emission channel (presented in red pseudocolor). The respective overlay images are shown on the right. No external BV was added to any cells presented in this Figure.

The iRFP713/V256C variant exhibits the highest quantum yield and molecular brightness among available BphP-based NIR FPs^2^,^3^ (Table 1); thus, we were curious about its properties in mammalian cells. Flow cytometry analysis (Fig. 6(a,b)) showed that the effective brightness of iRFP713/V256C-expressing cells was 150% compared to iRFP682- and iRFP713-expressing cells, which had brightnesses of 105% and 100%, respectively (Fig. 6(c)). The difference in brightness between iRFP682 and iRFP713/V256C cells was also observed by epifluorescence microscopy: the cells expressing iRFP713/V256C appeared brighter than iRFP682 cells for equal exposure times (Fig. 6(d)).

We next tested whether we could spectrally distinguish iRFP713/V256C, iRFP682 and iRFP713 variants in mammalian cells. We first performed flow cytometry of HeLa cells using a single excitation laser (640 nm) and two emission channels, far-red (670/30 nm) and NIR (710/20 nm) (Fig. 6(b)). As expected, the iRFP713/V256C-expressing cells were barely separable from iRFP682 cells due to the high spectral similarity (Table 1). In contrast, the iRFP713/V256C cells were easily distinguishable from the iRFP713-expressing cells (Fig. 6(b)).

We next performed imaging of the cells cytoplasmically expressing either iRFP713/V256C or iRFP713 using two filter sets. In the co-cultured cells iRFP713/V256C fluorescence was observed primarily in the far-red filter set (ex. 605/40 nm, em. 667/30 nm), whereas iRFP713 fluorescence was mainly detectable in the NIR filter set (ex. 682/12 nm, em. 721/42 nm) (Fig. 6(e)). We further co-expressed iRFP713/V256C and iRFP713 in the same cells. For that we fused iRFP713/V256C to a mitochondrial targeting signal and iRFP713 to a nuclear localization sequence. Again, the fusion proteins targeted to different cellular compartments were easily spectrally distinguishable (Fig. 6(f)). These experiments demonstrated that original iRFP713 and its bright blue-shifted iRFP713/V256C mutant can be used for two-color live cell imaging.

## Summary

We have characterized the BV interaction with dimeric BphP-derived NIR FPs. Our data show that the covalent binding of the BV chromophore in one monomer of a dimeric NIR FP bearing Cys residues either in the GAF domains only or in the PAS domains only may allosterically affect the interaction of BV with the other monomer. The substitution near the chromophore (e.g., at position 204) may further contribute to this effect. The presence of Cys15 in the PAS domains, in addition to Cys256 in the GAF domains, helps to overcome the allosteric inhibition and promotes the covalent binding of BV to Cys256 in both monomers. Therefore, the NIR FP variants with the Cys residues in both domains exhibit the narrow blue-shifted spectra as well as the highest fluorescence quantum yield and the highest molecular brightness. Moreover, the iRFP713/V256C protein with two Cys residues designed in this paper has the highest effective brightness when expressed in mammalian cells and should become the probe of choice for two-color NIR fluorescence imaging.

## Methods

### Plasmids, mutagenesis, protein expression and purification

The iRFP670, iRFP682 and iRFP713 genes were amplified and cloned into a pBAD/His-B vector (Invitrogen) using *Bgl*II and *Eco*RI sites. The iRFP670/C15S, iRFP670/C256S and iRFP670/C15S/C256S, iRFP682/C15S, iRFP682/C256S and iRFP682/C15S/C256S, iRFP713/T204A, iRFP713/C15S, iRFP713/C15S/V256C, iRFP713/V256C and iRFP713/C15S/V256C/T204A variants were obtained by site-directed mutagenesis using a QuickChange Mutagenesis Kit (Stratagene).

The iRFP670, iRFP682, iRFP713 proteins and their variants with polyhistidine tags on the N-termini were expressed in LMG194 host cells (Invitrogen) containing a pWA23h plasmid encoding heme oxygenase under the rhamnose promoter^3^. To initiate protein expression, bacterial cells were grown in RM medium supplemented with ampicillin, kanamycin and 0.02% rhamnose for 5 h at 37 °C. Then 0.002% arabinose was added, and cell culture was incubated for additional 12 h at 37 °C followed by 24 h at 18 °C. Proteins were purified using Ni-NTA agarose (Qiagen). The Ni-NTA elution buffer contained no imidazole and 100 mM EDTA. The elution buffer was substituted with PBS buffer using PD-10 desalting columns (GE Healthcare). The final purification was performed with ion-exchange chromatography using a MonoQ column (GE Healthcare).

### Spectral and biochemical characterization of purified proteins

Absorption spectra were recorded using a U-3900H spectrophotometer (Hitachi). The experiments were performed in 101.016-QS microcells (5 × 5 mm; Hellma) with path length of 5 mm at room temperature. The fluorescence experiments were conducted using a Cary Eclipse spectrofluorometer with FLR cells (10 × 10 × 4 mm; Agilent Technologies) with path length of 10 mm.

The extinction coefficient was calculated from a comparison of the absorbance values at the main peak with the absorbance value at the smaller peak at ~390 nm, assuming the latter had extinction coefficient of free BV of 39,900 M^−1^cm^−1^. To determine quantum yield, we compared the fluorescence signal of a purified protein to that of an equally absorbing Nile blue dye. The pH titrations were done using a series of buffers consisting of 100 mM sodium acetate, 300 mM NaCl for pH 2.5–5.0 and 100 mM NaH_2_PO_4_, 300 mM NaCl for pH 4.5–9.0 at room temperature. For chromophore binding assay purified samples were separated by SDS/PAGE in presence of 1 mM ZnCl_2_ and analyzed for zinc-induced fluorescence and then stained with Coomassie Blue.

Gel filtration of NIR FPs were performed on a Superose 12PC 3.2/30 column (GE Healthcare) using an AKTApurifier system (GE Healthcare). The samples of NIR FPs were prepared in buffer consisting of 50 mM NaH_2_PO_4_ and 150 mM NaCl at pH 8.0. 10 μl of each protein sample of 0.5 mg/ml was loaded on the column equilibrated with the same buffer. The protein chromatography standards (GE Healthcare) were used to evaluate a protein molecular weight.

### Analysis of protein 3D structure

The X-ray data of the PAS-GAF domains of the bacterial phytochrome *Rp*BphP2 (4E04.ent file^21^) deposited in PDB^25^ was used to analyze the distances between the chromophore and cysteine residues in the iRFP proteins.

### Protein unfolding assay

The protein unfolding was initiated by mixing 50 μl of the native protein with 500 μl of a buffer solution containing the desired concentration of GdnHCl (Nacalai Tesque). The concentration of the stock GdnHCl solution was determined by the refraction coefficient. The dependences of the chromophore absorbance, fluorescence and ellipticity at 222 nm on the GdnHCl concentration for the iRFP670, iRFP682 and iRFP713 variants were recorded at 23 °C after protein incubation in a solution of an appropriate denaturant concentration at 23 °С for 24 h. Further increases in the equilibration time did not result in noticeable changes in the detected characteristics.

The recorded fluorescence intensity was corrected for the total optical density of the solution (*D*_*Σ*_). The corrected fluorescence intensity was defined as 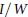, where 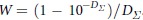. For details on the correction and normalization, please refer to^30^,^31^. These studies showed that 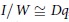, where *D* and *q* are the absorption and quantum yield of the fluorophore, respectively. Only the corrected fluorescence intensity can be used to evaluate the fraction of molecules that are in different structural states.

### Circular dichroism measurements

The circular dichroism (CD) spectra were obtained using a Jasco-810 spectropolarimeter (Jasco). The far-UV CD spectra were recorded in a 1 mm path length cell from 190 to 260 nm, with a step size of 0.1 nm. Three scans were averaged for all of the spectra. The CD spectra of the buffer solutions were recorded and subtracted from the protein spectra.

### Fitting of denaturation curves

The equilibrium dependences of the ellipticity at 222 nm on the GdnHCl concentration were fit using a two-state model:


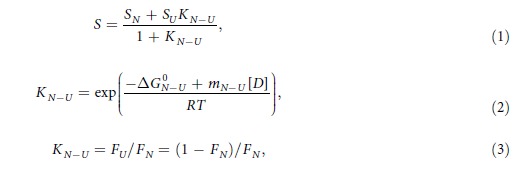


taking into account


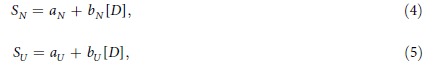


where *S* is the ellipticity at 222 nm at the measured GdnHCl concentration; [*D*] is the guanidine concentration; *m* is the linear dependence of Δ*G*_*N-U*_ on the denaturant concentration; 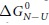
is the free energy of unfolding at 0 M denaturant; *F*_N_ and *F*_U_ are the fractions of native and unfolded molecules, respectively; and *S*_*N*_ and *S*_*U*_ are the signal of the native and unfolded states, respectively; *a*_*N*_, *b*_*N*_, *a*_*U*_ and *b*_*U*_ are constants. Fitting was performed using a nonlinear regression with Sigma Plot.

### Expression in mammalian cells

To construct mammalian expression plasmids, piRFP670, piRFP682, piRFP670/C15S, piRFP670/C256S, piRFP670/C15S/C256S, piRFP682/C15S, piRFP682/C256S, piRFP682/C15S/C256S, piRFP713 and piRFP713/V256C the respective genes were PCR-amplified as *Age*I-*Not*I fragments and swapped with a gene encoding EGFP in the pEGFP-N1 plasmid (Clontech). To generate the pNLS-iRFP713 and pMito-iRFP713/V256C plasmids, the *AgeI-NotI* fragments containing the iRFP713 and iRFP713/V256C genes were swapped with an ECFP gene in the pNLS-ECFP and pMito-ECFP plasmids (Clontech), respectively.

HeLa cells were grown in DMEM medium supplemented with 10% FBS, 0.5% penicillin-streptomycin and 2 mM glutamine (Life Technologies/Invitrogen). For fluorescence microscopy, cells were cultured in 35 mm glass-bottom Petri dishes with no. 1 coverglass (MatTek). Plasmid transfections were performed using an Effectene reagent (Qiagen).

### Flow cytometry

Flow cytometry analysis was performed using a BD LSRII flow cytometer. To detect HeLa cells co-transfected with EGFP and NIR FP, a 488 nm Ar gas laser line, a 640 nm solid-state laser and the 530/40 nm, 670/30 nm and 710/20 nm emission filters were used. To quantify cell fluorescence, the mean fluorescence intensity of the double-positive population in the NIR channel was divided by the mean fluorescence intensity of the same population in the green channel, thus normalizing the NIR signal to the transfection efficiency.

Discrimination of three types of HeLa cells co-expressing EGFP with iRFP670, iRFP713 and iRFP713/V256C was performed using a BD LSRII flow cytometer with a 488 nm laser and a 530/30 nm emission filter, as well as with a 640 nm laser and the 670/30 nm and 710/20 nm emission filters. For each cell type, 50,000 events were analyzed under the same conditions in a single experiment. The obtained dot plots were superimposed.

### Fluorescence microscopy

Epifluorescence microscopy of live HeLa cells was performed 48 h after the transfection. HeLa cells were imaged using an Olympus IX81 inverted epifluorescence microscope equipped with a 200 W Me-Ha arc lamp (Lumen220Pro, standardly equipped with a 780 nm cold mirror; Prior), the 40 × 1.2 NA oil and 100 × 1.4 NA oil immersion objective lens (UPlanSApo, Olympus), and two filter sets (605/40 nm excitation and 667/30 nm emission, and 682/12 nm excitation and 725/50 nm emission) (Chroma). SlideBook v.4.1 software (Intelligent Imaging Innovations) was used to operate the microscope.

## Additional Information

**How to cite this article**: Stepanenko, O. V. *et al.* Allosteric effects of chromophore interaction with dimeric near-infrared fluorescent proteins engineered from bacterial phytochromes. *Sci. Rep.* **6**, 18750; doi: 10.1038/srep18750 (2016).

## Supplementary Material

Supplementary Information

## Figures and Tables

**Table 1:** The spectral and biochemical properties of the iRFP670, iRFP682, iRFP713 proteins and their variants with different location of the Cys residues.

NIR FP group	Localization of Cys capable to bind BV	NIR FP	Absorbance maximum (nm)	FWHM of absorption spectrum (nm)	Emission maximum (nm)	FWHM of emission spectrum (nm)	Extinction coefficient (M^−1^cm^−1^)	Quantum yield (%)	Molecular brightness^a^ *vs.* iRFP713 (%)	p*K*a value	Effective brightness in mammalian cells^b^ *vs.* iRFP713 (%)
I	Cys15 in PAS	iRFP713	692	66	713	38	98,000	6.3	100	4.5	100
		iRFP713/T204A	696	59	720	39	86,000	5.1	71	n.d.	n.d.
		iRFP682/C256S	694	77	713	42	89,500	3.2	46	4.9	88
		iRFP670/C256S	675	82	702	48	92,900	5.7	86	4.3	103
II	no both Cys	iRFP713/C15S	685	80	710	42	68,000	5.5	61	n.d.	n.d.
		iRFP682/C15S/C256S	689	83	712	42	45,400	2.2	16	5.5	0.4
		iRFP670/C15S/C256S	671	81	699	48	76,300	4.6	57	5.3	0.3
II	Cys256 in GAF	iRFP713/C15S/V256C	665	97	676	67	66,000	7.2	77	n.d.	n.d.
		iRFP713/C15S/V256C/T204A	659	106	681	57	51,000	6.2	51	n.d.	n.d.
		iRFP682/C15S	659	111	683	60	53,800	5.0	44	5.0	27
		iRFP670/C15S	641	103	669	69	85,400	10.3	143	4.7	34
IV	Cys15 in PAS and Cys256 in GAF	iRFP713/V256C	662	66	680	46	94,000	14.5	221	4.5	150
		iRFP682	663	69	682	42	90,000	11.1	162	4.5	105
		iRFP670	644	78	670	42	110,000	12.2	217	4.0	119

^a^Molecular brightness is a product of extinction coefficient and fluorescence emission quantum yield.

^b^Determined as a mean NIR fluorescence intensity of HeLa cells with no supply of exogenous BV after normalization to a mean fluorescence intensity of co-transfected EGFP. FWHM, full width at half maximum of spectrum. n.d., not determined.

## References

[b1] Piatkevich K. D., Subach F. V. & Verkhusha V. V. Engineering of bacterial phytochromes for near-infrared imaging, sensing, and light-control in mammals. Chem Soc Rev 42, 3441–3452 (2013). 23361376 10.1039/c3cs35458j PMC3618476

[b2] Shcherbakova D. M., Baloban M. & Verkhusha V. V. Near-infrared fluorescent proteins engineered from bacterial phytochromes. Curr Opin Chem Biol 27, 52–63 (2015). 26115447 10.1016/j.cbpa.2015.06.005 PMC4553112

[b3] Shcherbakova D. M. & Verkhusha V. V. Near-infrared fluorescent proteins for multicolor *in vivo* imaging. Nat Methods 10, 751–754 (2013). 23770755 10.1038/nmeth.2521 PMC3737237

[b4] Yu D. *et al.* An improved monomeric infrared fluorescent protein for neuronal and tumour brain imaging. Nat Commun 5, 3626 (2014). 24832154 10.1038/ncomms4626 PMC4077998

[b5] Bhattacharya S., Auldridge M. E., Lehtivuori H., Ihalainen J. A. & Forest K. T. Origins of fluorescence in evolved bacteriophytochromes. J Biol Chem 289, 32144–32152 (2014). 25253687 10.1074/jbc.M114.589739 PMC4231690

[b6] Auldridge M. E., Satyshur K. A., Anstrom D. M. & Forest K. T. Structure-guided engineering enhances a phytochrome-based infrared fluorescent protein. J Biol Chem 287, 7000–7009 (2012). 22210774 10.1074/jbc.M111.295121 PMC3293566

[b7] Filonov G. S. *et al.* Bright and stable near-infrared fluorescent protein for *in vivo* imaging. Nat Biotechnol 29, 757–761 (2011). 21765402 10.1038/nbt.1918 PMC3152693

[b8] Piatkevich K. D., Subach F. V. & Verkhusha V. V. Far-red light photoactivatable near-infrared fluorescent proteins engineered from a bacterial phytochrome. Nat Commun 4, 2153 (2013). 23842578 10.1038/ncomms3153 PMC3749836

[b9] Filonov G. S. & Verkhusha V. V. A Near-Infrared BiFC Reporter for *In Vivo* Imaging of Protein-Protein Interactions. Chem Biol 20, 1078–1086 (2013). 23891149 10.1016/j.chembiol.2013.06.009 PMC3757571

[b10] Chen M. *et al.* Novel near-infrared BiFC systems from a bacterial phytochrome for imaging protein interactions and drug evaluation under physiological conditions. Biomaterials 48, 97–107 (2015). 25701035 10.1016/j.biomaterials.2015.01.038

[b11] Burgie E. S. & Vierstra R. D. Phytochromes: An Atomic Perspective on Photoactivation and Signaling. The Plant cell 26, 4568–4583 (2014). 25480369 10.1105/tpc.114.131623 PMC4311201

[b12] Rockwell N. C. & Lagarias J. C. A brief history of phytochromes. Chemphyschem 11, 1172–1180 (2010). 20155775 10.1002/cphc.200900894 PMC2880163

[b13] Bhoo S. H., Davis S. J., Walker J., Karniol B. & Vierstra R. D. Bacteriophytochromes are photochromic histidine kinases using a biliverdin chromophore. Nature 414, 776–779 (2001). 11742406 10.1038/414776a

[b14] Lamparter T. *et al.* The biliverdin chromophore binds covalently to a conserved cysteine residue in the N-terminus of Agrobacterium phytochrome Agp1. Biochemistry 43, 3659–3669 (2004). 15035636 10.1021/bi035693l

[b15] Lamparter T. *et al.* Biliverdin binds covalently to agrobacterium phytochrome Agp1 via its ring A vinyl side chain. J Biol Chem 278, 33786–33792 (2003). 12824166 10.1074/jbc.M305563200

[b16] Davis S. J., Kurepa J. & Vierstra R. D. The Arabidopsis thaliana HY1 locus, required for phytochrome-chromophore biosynthesis, encodes a protein related to heme oxygenases. Proc Natl Acad Sci U S A 96, 6541–6546 (1999). 10339624 10.1073/pnas.96.11.6541 PMC26918

[b17] Yeh K. C., Wu S. H., Murphy J. T. & Lagarias J. C. A cyanobacterial phytochrome two-component light sensory system. Science 277, 1505–1508 (1997). 9278513 10.1126/science.277.5331.1505

[b18] Wu S. H. & Lagarias J. C. Defining the bilin lyase domain: lessons from the extended phytochrome superfamily. Biochemistry 39, 13487–13495 (2000). 11063585 10.1021/bi001123z

[b19] Gambetta G. A. & Lagarias J. C. Genetic engineering of phytochrome biosynthesis in bacteria. Proc Natl Acad Sci U S A 98, 10566–10571 (2001). 11553807 10.1073/pnas.191375198 PMC58506

[b20] Moffat K. Time-resolved crystallography and protein design: signalling photoreceptors and optogenetics. Philos Trans R Soc Lond B Biol Sci 369, 20130568 (2014). 24914168 10.1098/rstb.2013.0568 PMC4052877

[b21] Bellini D. & Papiz M. Z. Dimerization properties of the RpBphP2 chromophore-binding domain crystallized by homologue-directed mutagenesis. Acta Crystallogr D Biol Crystallogr 68, 1058–1066 (2012). 22868772 10.1107/S0907444912020537

[b22] Auldridge M. E. & Forest K. T. Bacterial phytochromes: more than meets the light. Crit Rev Biochem Mol Biol 46, 67–88 (2011). 21250783 10.3109/10409238.2010.546389

[b23] Wagner J. R. *et al.* Mutational analysis of Deinococcus radiodurans bacteriophytochrome reveals key amino acids necessary for the photochromicity and proton exchange cycle of phytochromes. J Biol Chem 283, 12212–12226 (2008). 18192276 10.1074/jbc.M709355200 PMC2431007

[b24] Stepanenko O. V. *et al.* A knot in the protein structure - probing the near-infrared fluorescent protein iRFP designed from a bacterial phytochrome. FEBS J 281, 2284–2298 (2014). 24628916 10.1111/febs.12781 PMC4009348

[b25] Dutta S. *et al.* Data deposition and annotation at the worldwide protein data bank. Mol Biotechnol 42, 1–13 (2009). 19082769 10.1007/s12033-008-9127-7

[b26] Borucki B. *et al.* Mechanism of Cph1 phytochrome assembly from stopped-flow kinetics and circular dichroism. Biochemistry 42, 13684–13697 (2003). 14622015 10.1021/bi035511n

[b27] Narikawa R. *et al.* Structures of cyanobacteriochromes from phototaxis regulators AnPixJ and TePixJ reveal general and specific photoconversion mechanism. Proc Natl Acad Sci U S A 110, 918–923 (2013). 23256156 10.1073/pnas.1212098110 PMC3549089

[b28] Hauck A. F. *et al.* The photoinitiated reaction pathway of full-length cyanobacteriochrome Tlr0924 monitored over 12 orders of magnitude. J Biol Chem 289, 17747–17757 (2014). 24817121 10.1074/jbc.M114.566133 PMC4067208

[b29] Shcherbakova D. M. *et al.* Molecular basis of spectral diversity in near-infrared phytochrome-based fluorescent proteins. Chem Biol 22, 1540–1551 (2015). 26590639 10.1016/j.chembiol.2015.10.007 PMC4667795

[b30] Kuznetsova I. M., Sulatskaya A. I., Povarova O. I. & Turoverov K. K. Reevaluation of ANS Binding to Human and Bovine Serum Albumins: Key Role of Equilibrium Microdialysis in Ligand - Receptor Binding Characterization. PLoS One 7, e40845 (2012). 22829890 10.1371/journal.pone.0040845 PMC3400656

[b31] Fonin A. V., Sulatskaya A. I., Kuznetsova I. M. & Turoverov K. K. Fluorescence of dyes in solutions with high absorbance. Inner filter effect correction. PLoS One 9, e103878 (2014). 25072376 10.1371/journal.pone.0103878 PMC4114876

