# Supplementary material for: Allosteric effects of chromophore interaction with dimeric near-infrared fluorescent proteins engineered from bacterial phytochromes
Source: Sci Rep. 2016 Jan 4;6:18750. doi: 10.1038/srep18750 (PMC4698714; doi:10.1038/srep18750)
Supplement: Supplementary Information [file srep18750-s1.pdf]

# Allosteric effects of chromophore interaction with dimeric near-infrared fluorescent proteins engineered from bacterial phytochromes

**Olesya V. Stepanenko, Mikhail Baloban, Grigory S. Bublikov, Daria M. Shcherbakova, Olga V. Stepanenko, Konstantin K. Turoverov, Irina M. Kuznetsova and Vladislav V. Verkhusha**

## Supplementary Information

## Supplementary Figures

**Supplementary Figure 1.** Alignment of amino acid sequences of iRFP670, iRFP682 and iRFP713 with their wild-type templates *Rp*BphP6-PAS-GAF and *Rp*BphP2-PAS-GAF.

**Supplementary Figure 2.** SDS-PAGE gel with Coomassie Blue (CB) and $ZnCl_2$ (Zn) staining of the purified iRFP670, iRFP682, iRFP713 and their mutants.

**Supplementary Figure 3.** Gel filtration of iRFP670, iRFP682 and iRFP713/V256C**.**

**Supplementary Figure 4.** Spectral properties of iRFP713, iRFP682, iRFP670 and their mutants.

**Supplementary Figure 5.** Proposed BV adducts in the iRFP variants having either Cys15 in PAS domain or/and Cys256 in GAF domain.

**Supplementary Figure 6.** Changes in absorption of iRFP713 variants in GdnHCl.

**Supplementary Figure 7.** Stability of iRFP670, iRFP682 and iRFP713 variants in GdnHCl.

**Supplementary Figure 8.** Brightness of iRFP670, iRFP682 and their mutants in mammalian cells.

## Supplementary Tables

**Supplementary Table 1.** Midpoints of GdnHCl-induced denaturation of iRFP670, iRFP682, iRFP713 and their variants with different location of Cys residues.

```
                          1        10         20         30         40         50         60
RpBphP2(4E04) MTEGSVARQP DLSTCDDEPI HIPGAIQPHG LLLALAADMT IV-AGSDNLP ELTGLAIGAL
RpBphP2       MTEGSVARQP DLSTCDDEPI HIPGAIQPHG LLLALAADMT IV-AGSDNLP ELTGLAIGAL
iRFP713       MAEGSVARQP DLLTCDDEPI HIPGAIQPHG LLLALAADMT IV-AGSDNLP ELTGLAIGAL
iRFP682       MAEGSVARQP DLLTCDDEPI HIPGAIQPHG LLLALAADMT IV-AGSDNLP ELTGLAIGAL
RpBphP6       MPRK-----V DLTSCDREPI HIPGSIQPCG CLLACDAQAV RITRISENAG AFFGRETPRV
iRFP670       MARK-----V DLTSCDREPI HIPGSIQPCG CLLACDAQAV RITRITENAG AFFGRETPRV
              ---------- ---------- ---------- ---------- ---------- ----------
                                     PAS

                                 70         80         90        100        110        120
RpBphP2(4E04) IGRSAADVFD SETHNRLTIA LAEPGAAVGA PIAVGFTMPD GERAFNGSWH RHDQLVFLEL
RpBphP2       IGRSAADVFD SETHNRLTIA LAEPGAAVGA PIAVGFTMRK DAGFV-GSWH RHDQLVFLEL
iRFP713       IGRSAADVFD SETHNRLTIA LAEPGAAVGA PITVGFTMRK DAGFI-GSWH RHDQLIFLEL
iRFP682       IGRSAADVFD SETHNRLTIA LAEPGAAVGA PITVGFTMRK DAGFI-GSWH RHDQLIFLEL
RpBphP6       GELLADYFGE TEAHALRNAL AQSSDPKRPA LIFGWRDGLT GRTFD-ISLH RHDGTSIVEF
iRFP670       GELLADYFGE TEAHALRNAL AQSSDPKRPA LIFGWRDGLT GRTFD-ISLH RHDGTSIIEF
              ---------- ---------- ---------- ---------- ---------- ----------
                                     PAS

                                130        140        150        160        170        180
RpBphP2(4E04) EPPQRDVRYP QAFFRSVRSA IRRLQAAETL ESACAAAAQE VREITGFDRV MIYRFASDFS
RpBphP2       EPPQRDVAEP QAFFRRTNSA IRRLQAAETL ESACAAAAQE VREITGFDRV MIYRFASDFS
iRFP713       EPPQRDVAEP QAFFRRTNSA IRRLQAAETL ESACAAAAQE VRKITGFDRV MIYRFASDFS
iRFP682       EPPQRDVAEP QAFFRRTNSA IRRLQAAETL ESACAAAAQE VRKITGFDRV MIYRFASDFS
RpBphP6       EPAAADQADN PL--RLTRQI IARTKELKSL EEMAARVPRY LQAMLGYHRV MMYRFADDGS
iRFP670       EPAAAEQADN PL--RLTRQI IARTKELKSL EEMAARVPRY LQAMLGYHRV MLYRFADDGS
              -------___ __________ __________ __________ __________ __________
              PAS    GAF

                                190        200        210        220        230        240
RpBphP2(4E04) GEVIAEDRCA EVESYLGLHF PASDIPAQAR RLYTINPVRI IPDINYRPVP VTPDLNPRTG
RpBphP2       GEVIAEDRCA EVESYLGLHF PASDIPAQAR RLYTINPVRI IPDINYRPVP VTPDLNPVTG
iRFP713       GEVIAEDRCA EVESKLGLHY PASTVPAQAR RLYTINPVRI IPDINYRPVP VTPDLNPVTG
iRFP682       GVVIAEDRCA EVESKLGLHY PASAVPAQAR RLYTINPVRI IPDINYRPVP VTPDLNPVTG
RpBphP6       GKVIGEAKRS DLESFLGQHF PASDIPQQAR LLYLKNAIRV ISDSRGISSR IVPERDAS-G
iRFP670       GMVIGEAKRS DLESFLGQHF PASLVPQQAR LLYLKNAIRV VSDSRGISSR IVPEHDAS-G
              __________ __________ __________ __________ __________ __________
                                     GAF

                                250        260        270        280        290        300
RpBphP2(4E04) RPIDLSFAIL RSVSPVHLEY MRNIGMHGTM SISILRGERL WGLIACHHRK PNYVDLEVRQ
RpBphP2       RPIDLSFAIL RSVSPVHLEY MRNIGMHGTM SISILRGERL WGLIACHHRK PNYVDLDGRQ
iRFP713       RPIDLSFAIL RSVSPVHLEF MRNIGMHGTM SISILRGERL WGLIVCHHRT PYYVDLDGRQ
iRFP682       RPIDLSFAIL RSVSPCHLEF MRNIGMHGTM SISILRGERL WGLIVCHHRT PYYVDLDGRQ
RpBphP6       AALDLSFAHL RSVSPIHLEY LRNMGVSASM SLSIIIDGTL WGLIACHHYE PRAVPMAQRV
iRFP670       AALDLSFAHL RSISPCHLEF LRNMGVSASM SLSIIIDGTL WGLIICHHYE PRAVPMAQRV
              __________ __________ __________ __________ __________ __________
                                     GAF

                                310        320
RpBphP2(4E04) ACELVAQVLA WQIGVMEEQA
RpBphP2       ACELVAQVLA WQIGVMEE
iRFP713       ACELVAQVLA WQIGVMEE
iRFP682       ACELVAQVLA WQIGVMEE
RpBphP6       AAEMFADFFS LHFTAAHHQR
iRFP670       AAEMFADFLS LHFTAAHHQR
              __________ __________
                       GAF
```

**Supplementary Figure 1. Alignment of amino acid sequences of iRFP670, iRFP682 and iRFP713 with their wild-type templates *Rp*BphP6-PAS-GAF and *Rp*BphP2-PAS-GAF.** The residues in red font are the conservative Cys residue (Cys15) in the PAS domain of all BphPs and the unique Cys residue (Cys256) in the GAF domain of iRFP670 and iRFP682.

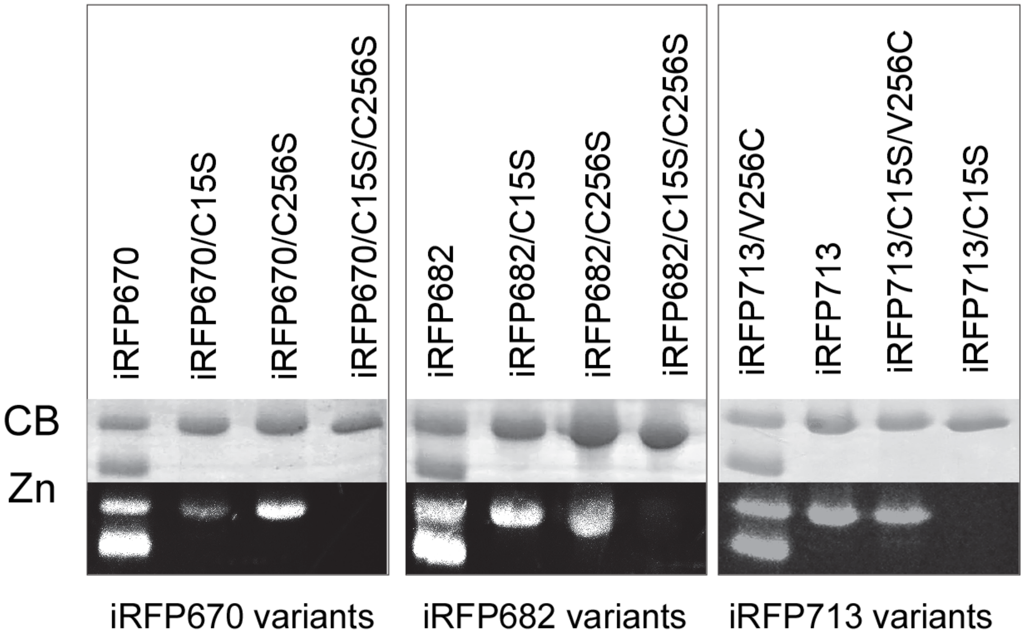


**Supplementary Figure 2. SDS-PAGE gel with Coomassie Blue (CB) and $ZnCl_2$ (Zn) staining of the purified iRFP670, iRFP682, iRFP713 and their mutants.** The Zn staining indicates the covalent binding of BV to all protein variants except those without Cys residues in both PAS and GAF domains.

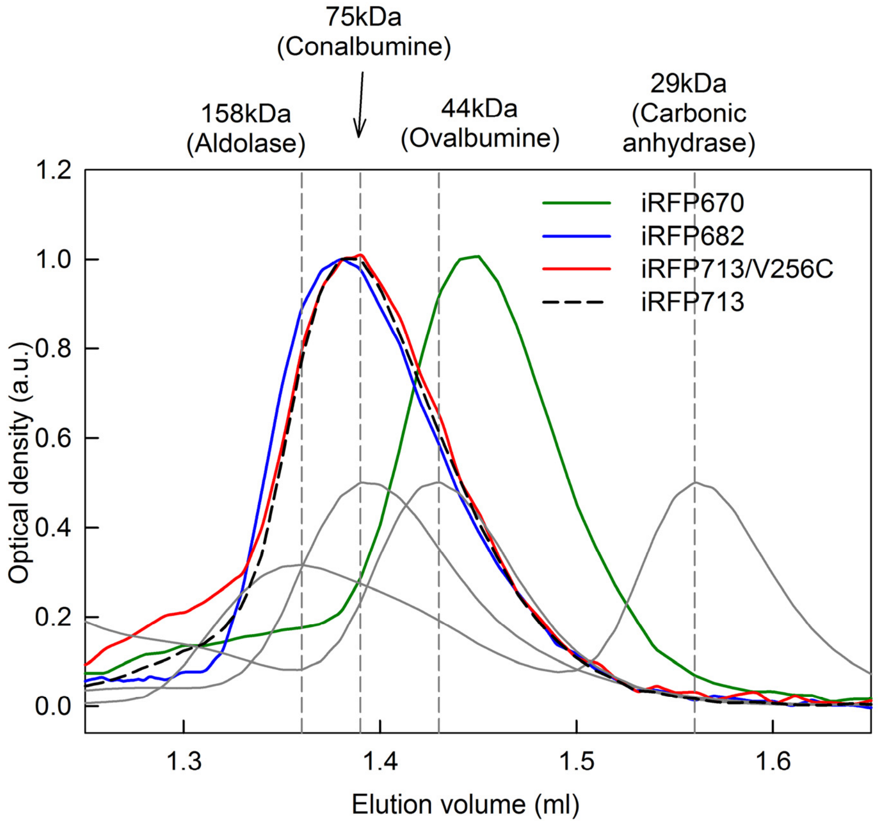


**Supplementary Figure 3. Gel filtration of iRFP670, iRFP682 and iRFP713/V256C.** The protein concentrations were 0.5 mg/ml. The iRFP682 (solid blue line) and iRFP713/V256C (solid red line) proteins were eluted as the single peaks corresponding to the protein dimers. At this protein concentration and gel filtration conditions (see Methods) iRFP670 (solid green line) had the elution profile corresponding to the monomer, in contrast to its dimeric behavior previously observed in cells (Shcherbakova and Verkhusha, 2013). The dimer of iRFP713 (dashed black line) and the protein chromatography standards (solid grey lines) were used to evaluate the molecular weight.

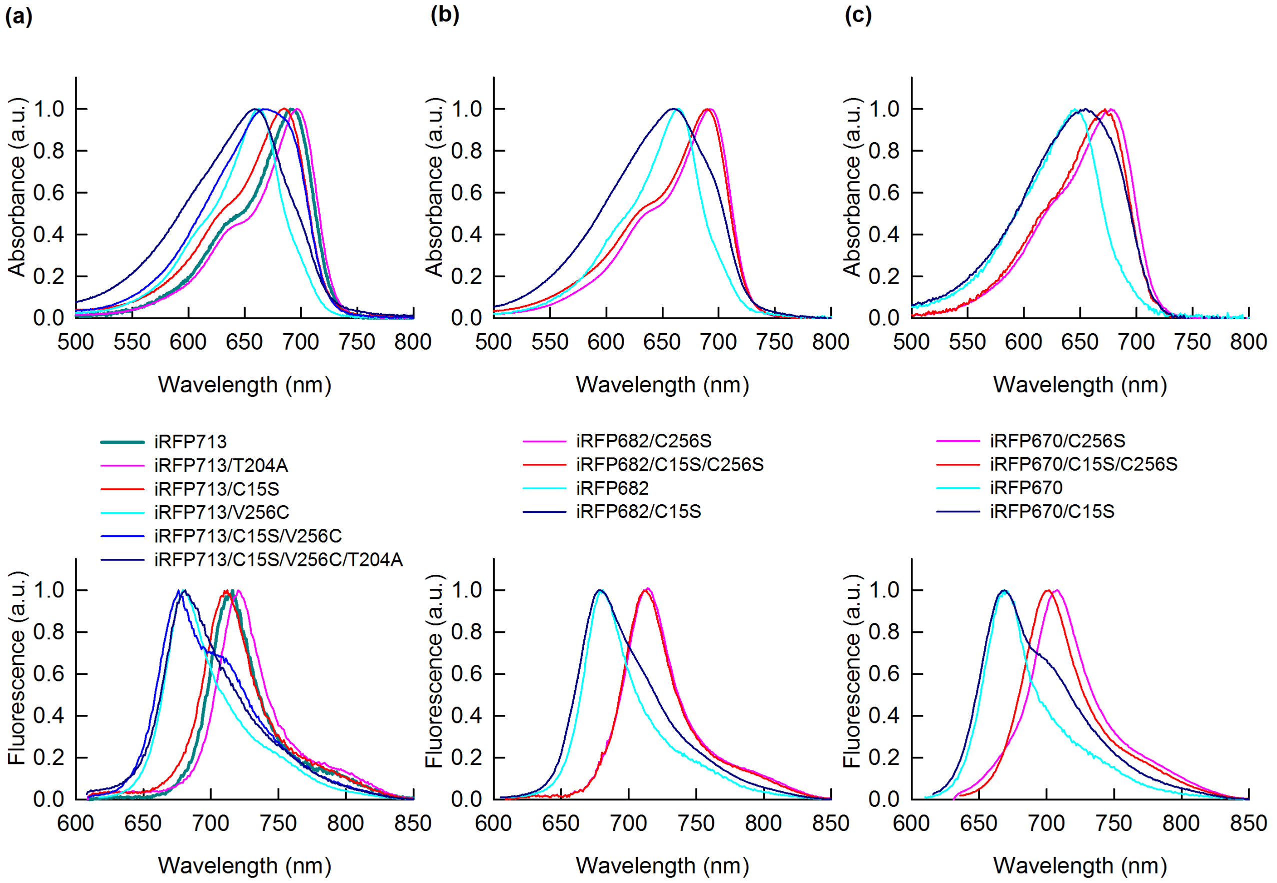


**Supplementary Figure 4. Spectral properties of iRFP713, iRFP682, iRFP670 and their mutants.** Absorption and fluorescence spectra of (**a**) iRFP713, (**b**) iRFP682 and (**c**) iRFP670 variants with Cys residues either in PAS or in GAF domains, with or without both conservative Cys residues.

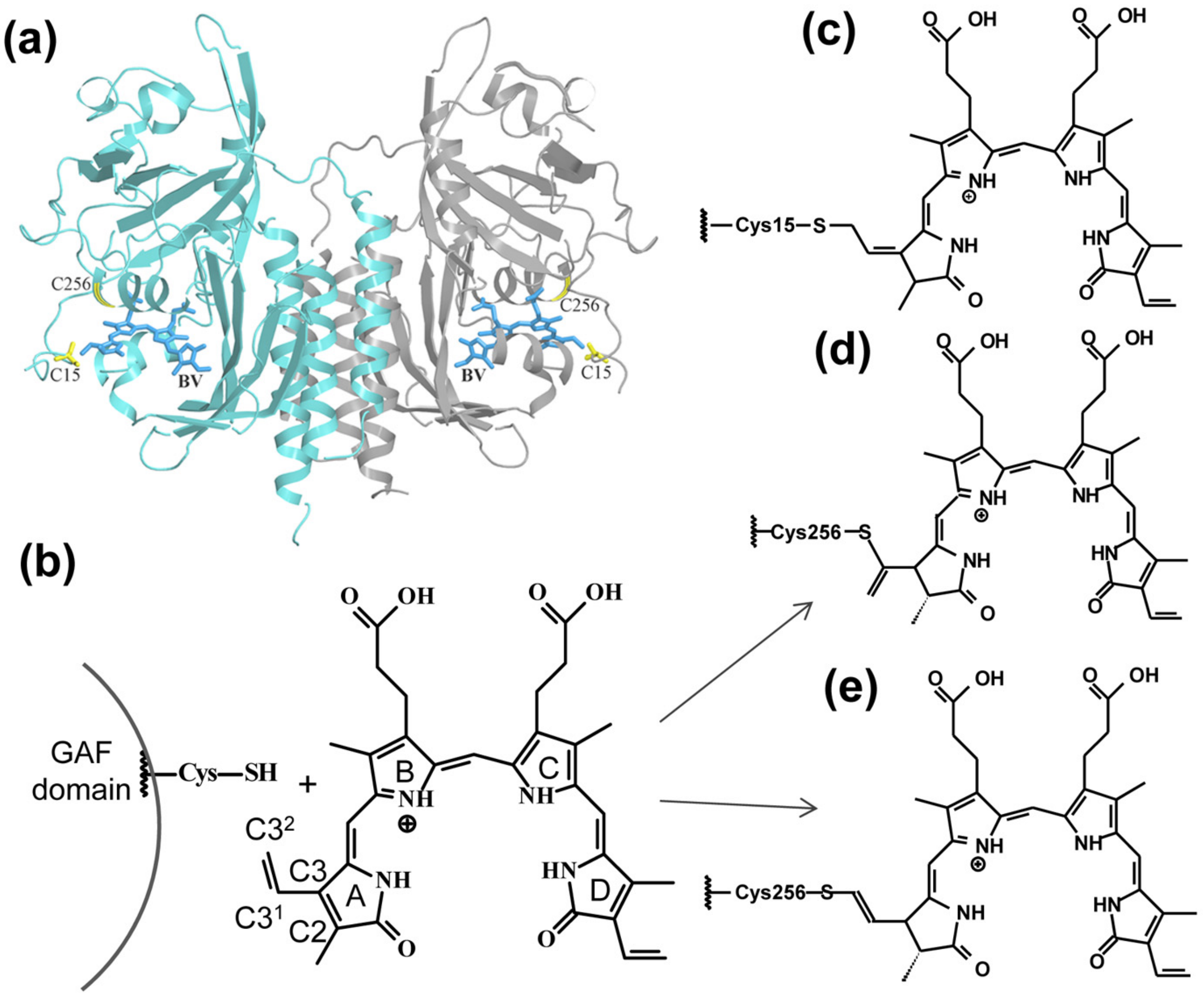


**Supplementary Figure 5. Proposed BV adducts in the iRFP variants having either Cys15 in PAS domain or/and Cys256 in GAF domain.** (**a**) Cys15 in PAS domain and Cys256 in GAF domain are mapped on the X-ray structure of RpBphP2-PAS-GAF protein (4E04 file in PDB). Cys residues are shown in yellow, and protein monomers in the dimer are marked in blue and gray. When free BV molecule with two double bonds in the pyrrole ring A covalently binds to Cys256 in the GAF domain, the spectral blue shift is observed with respect **(b)** to unbound BV and **(c)** to BV covalently bound to Cys15 in the PAS domain. (**d, e**) The blue spectral shift can result from the reduced length of the conjugated π-electron system due to BV-chromophore isomerization that leads to uncoupling of one double bond in the BV adducts from the rest of the conjugated electron system.

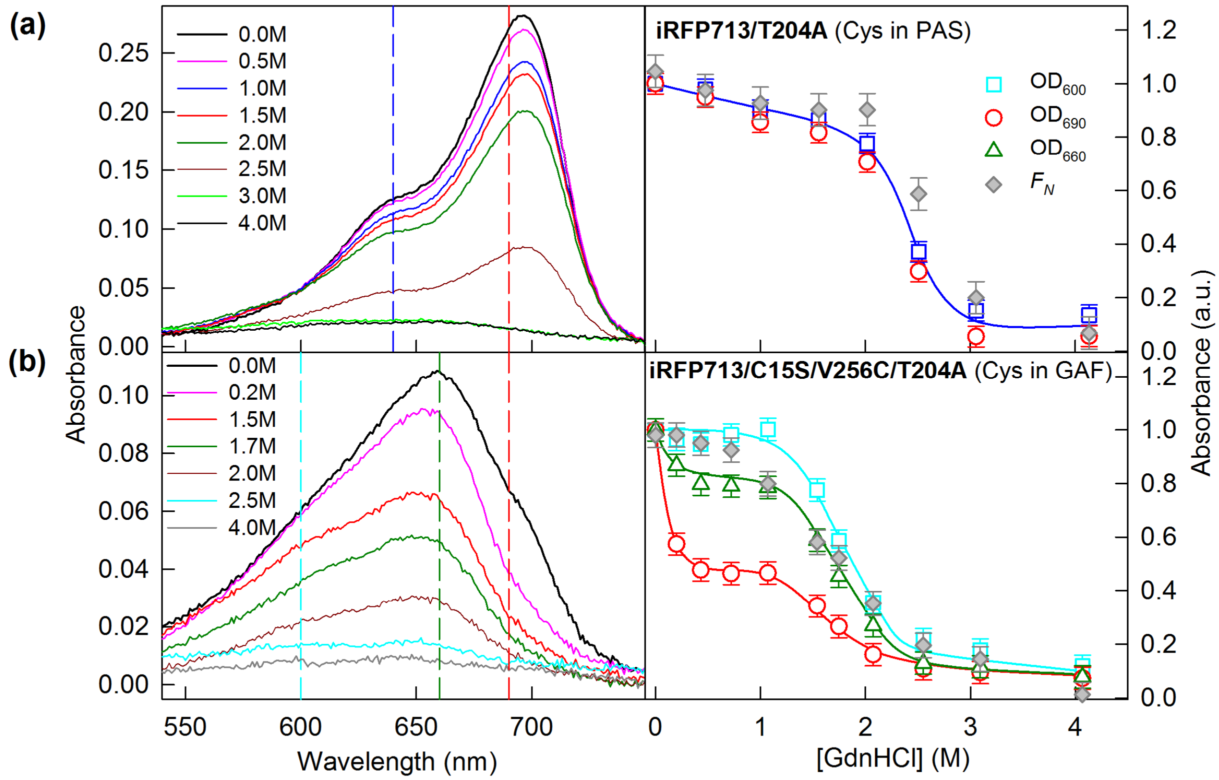


**Supplementary Figure 6. Changes in absorption of iRFP713 variants in GdnHCl.** The absorption spectra (left panels) and dependences of absorbance at 640 and 690 nm, or at 600, 660 and 690 nm (right panels) on GdnHCl concentration of **(a)** iRFP713/T204A and **(b)** iRFP713/C15S/V256C/T204A mutants are shown. The numbers on the curves indicate the denaturant concentration in the protein samples. Colored vertical dashed lines show the selected for further analysis wavelengths. The data were normalized to the absorption at corresponding wavelength of the iRFP713 variant in buffered solution (n=3; error bars are s.e.m.). The stability of the protein structure against GdnHCl-induced unfolding is shown as the dependences of the part of native molecules $F_N$ on GdnHCl concentration (gray symbols). $F_N$ was calculated on the basis of ellipticity at 222 nm.

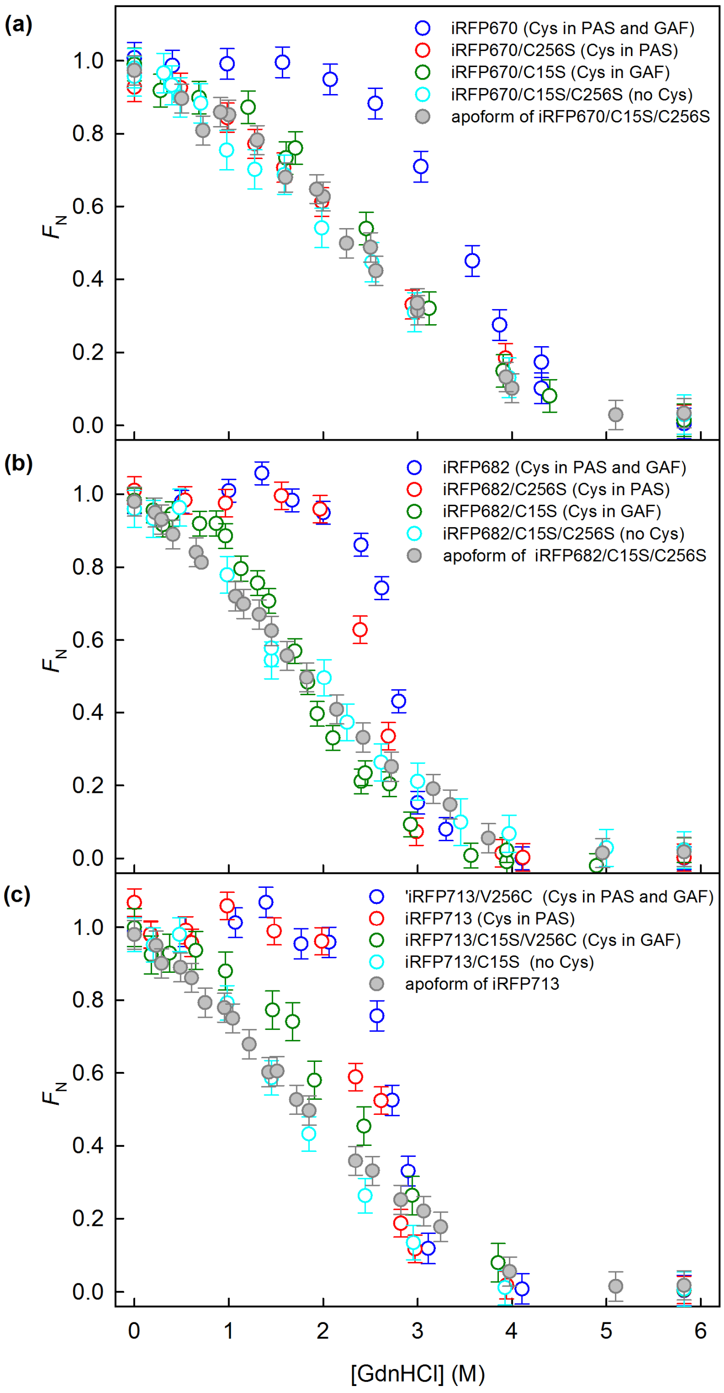


**Supplementary Figure 7. Stability of iRFP670, iRFP682 and iRFP713 variants in GdnHCl.** Stability of **(a)** iRFP670, **(b)** iRFP682 and **(c)** iRFP713 variants with Cys in GAF or PAS domains, without or with both conservative Cys residues. The part of native molecules $F_N$ was calculated on the basis of ellipticity at 222 nm. For comparison the data of iRFP670 apoprotein (gray circles) are shown (n=3; error bars are s.e.m.).

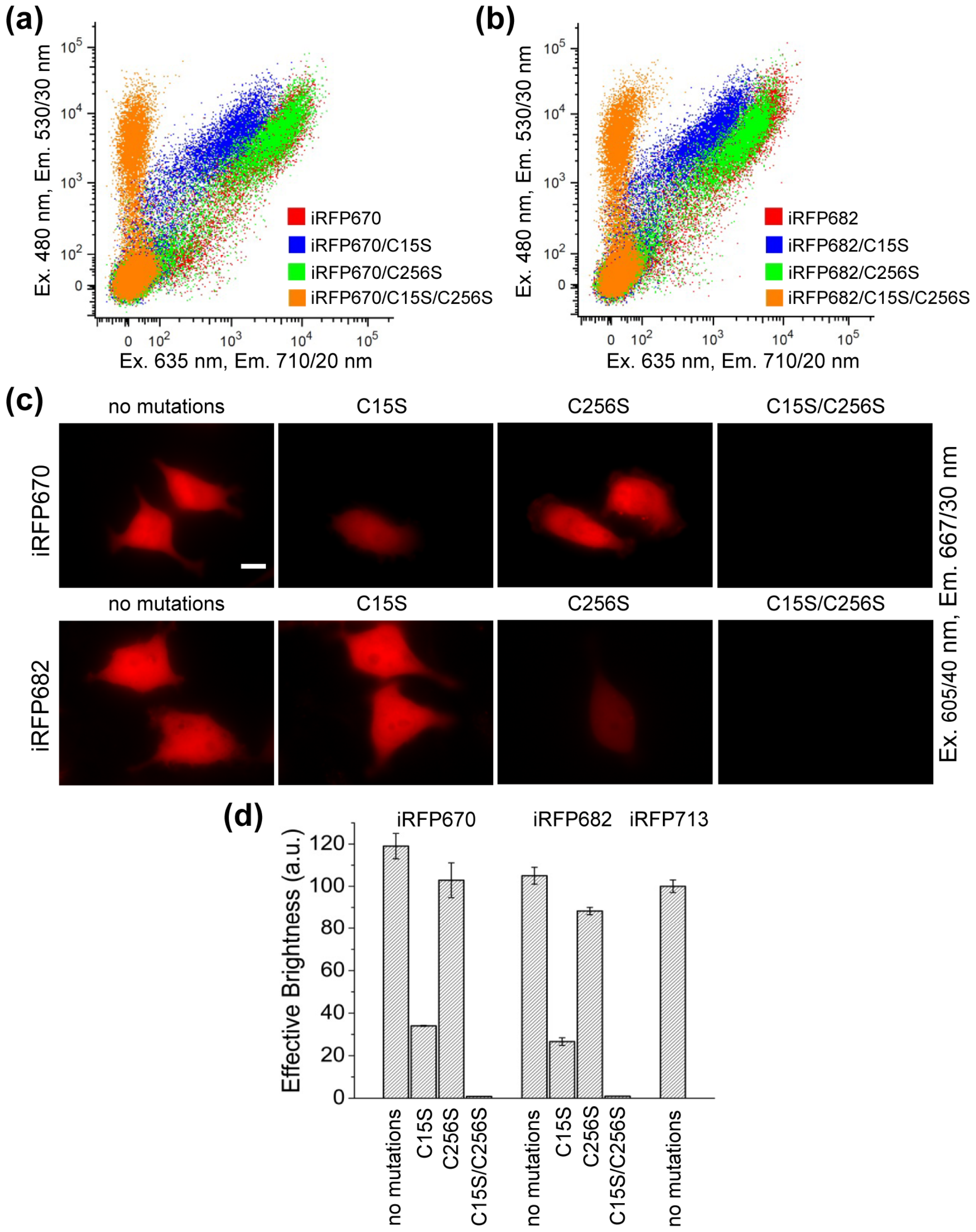


**Supplementary Figure 8. Brightness of iRFP670, iRFP682 and their mutants in mammalian cells.** (**a**) HeLa cells were co-transfected with one of the iRFP670 variants and EGFP as the transfection efficiency control and analyzed in NIR (x axis: excitation at 635 nm and emission at 710/20 nm) and green (y axis: excitation at 488 nm and emission at 530/30 nm) channels using flow cytometry. (**b**) The same as in (a) but transfected with the iRFP682 variants. (**c**) The representative images of HeLa cells transfected with iRFP670 variants (top row) and with iRFP682 variants (bottom row) are shown. The 605/40 nm excitation and 667/30 nm emission filters were used. The exposure time for cells expressing original iRFP670 was 8-fold shorter and for cells expressing original iRFP682 was 5-fold shorter than for all others. Scale bar is 10 μm. (**d**) NIR fluorescence of HeLa cells in (a,b) was normalized to that of EGFP intensity and to excitation and emission spectra of each of the iRFP variants. The normalized fluorescence intensities of the cells expressing an iRFP713 control (see Fig. 6) were assumed 100%.

**Supplementary Table 1. Midpoints of GdnHCl-induced denaturation of iRFP670, iRFP682, iRFP713 and their variants with different location of Cys residues.**

| NIR FP group | Localization of Cys capable to bind BV | NIR FP variant | $C_m$ of GdnHCl (M) |
|---|---|---|---|
| I | Cys15 in PAS | iRFP713 | 2.5 ± 0.1 |
| | | iRFP713/T204A | 2.6 ± 0.1 |
| | | iRFP682/C256S | 2.5 ± 0.1 |
| | | iRFP670/C256S | 2.4 ± 0.1 |
| | | | |
| II | no both Cys | iRFP713/C15S | 1.7 ± 0.2 |
| | | iRFP682/C15S/C256S | 1.8 ± 0.1 |
| | | iRFP670/C15S/C256S | 2.3± 0.1 |
| | | | |
| III | Cys256 in GAF | iRFP713/C15S/V256C | 2.3 ± 0.1 |
| | | iRFP713/C15S/V256C/T204A | 1.8 ± 0.1 |
| | | iRFP682/C15S | 1.8 ± 0.1 |
| | | iRFP670/C15S | 2.6 ± 0.1 |
| | | | |
| IV | Cys15 in PAS and Cys256 in GAF | iRFP713/V256C | 2.8 ± 0.1 |
| | | iRFP682 | 2.8 ± 0.1 |
| | | iRFP670 | 3.5 ± 0.1 |
| | | | |
| apoforms | no BV | iRFP713 | 1.8 ± 0.2 |
| | | iRFP682/C15S/C256S | 1.6 ± 0.1 |
| | | iRFP670/C15S/C256S | 2.3± 0.2 |

**Supplementary References.**

Shcherbakova, D. M. & Verkhusha, V. V. Near-infrared fluorescent proteins for multicolor *in vivo* imaging. *Nature Methods* **10**, 751-754 (2013).
